# IL-21 Is Required for Optimal Antibody Production and T Cell Responses during Chronic *Toxoplasma gondii* Infection

**DOI:** 10.1371/journal.pone.0062889

**Published:** 2013-05-07

**Authors:** Jason S. Stumhofer, Jonathan S. Silver, Christopher A. Hunter

**Affiliations:** 1 Department of Microbiology and Immunology, University of Arkansas for Medical Sciences, Little Rock, Arkansas, United States of America; 2 Department of Pathobiology, School of Veterinary Medicine, University of Pennsylvania, Philadelphia, Pennsylvania, United States of America; University of Oklahoma Health Sciences Center, United States of America

## Abstract

Previous studies have indicated that *Il21r* ^−/−^ mice chronically infected with *Toxoplasma gondii* display a defect in serum IgG; however, the basis for this antibody defect was not defined and questions remain about the role of IL-21 in promoting the production of IL-10, which is required to limit infection-induced pathology during toxoplasmosis. Therefore, *Il21* ^−/−^ mice were challenged with *T. gondii* to determine whether IL-21 impacts the parasite-specific CD8^+^ T cell response, its contribution to thymus-dependent antibody production after infection, and balance between protective and pathogenic responses. Whereas IL-21 has been implicated in the differentiation of IL-10 producing CD4^+^ T cells no immune-mediated pathology was evident in *Il21* ^−/−^ mice during the acute response, nor was there a defect in the development of this population in chronically infected *Il21* ^−/−^ mice. However, *Il21* ^−/−^ mice displayed a defect in IgG production after infection that correlated with a decrease in GC B cell numbers, the CD4^+^ and CD8^+^ T cell numbers in the brain were reduced over the course of the chronic infection leading to a decrease in total IFN-γ production and an increase in parasite numbers associated with susceptibility to toxoplasmic encephalitis. Together, these results identify a key role for IL-21 in shaping the humoral and cellular response to *T. gondii*, but indicate that IL-21 has a limited role in regulating immunopathology.

## Introduction

Infection with the protozoan parasite *Toxoplasma gondii* results in a persistent infection that affects the central-nervous system and is typically regarded as asymptomatic [1]. *T. gondii* has the ability to invade and replicate within host cells, and long-term resistance to this organism is dependent on the ability of CD8^+^ T cells to recognize and respond to infected cells through cytokine production or cytolysis, making them an integral part of the protective immune response against this organism. Thus, depletion of CD8^+^ T cells alone, but not CD4^+^ T cells, during chronic infection with *T. gondii* leads to increased susceptibility, emphasizing the importance of these lymphocytes in the local control of parasites in the brain [2]. Likewise, B cells also contribute to the control of this intracellular parasite and B cell deficient mice challenged with *T. gondii* succumb to disease between 3 to 4 weeks after infection. However, these mice can be rescued through administration of anti-*Toxoplasma* IgG antibody [3], indicating that B cell production of parasite specific antibodies contributes to the control of toxoplasmic encephalitis (TE).

While the cell-mediated immune response is essential for control of *T. gondii* in the brain, this response must be regulated in order to prevent damage by the immune response. In particular, the production of Interleukin-10 (IL-10) during chronic TE has an important role in limiting pathology as several studies have suggested that in its absence [4], or when its production is impaired [5], a lethal inflammatory response ensues in the brain characterized by increased numbers of CD4^+^ T cells and elevated production of inflammatory cytokines. One cytokine that is involved in the induction of IL-10 by CD4^+^ T cells is IL-27; however, it is unclear whether this is a direct effect of IL-27 on CD4^+^ T cells, or an indirect effect through IL-27 mediated induction of IL-21, which then drives IL-10 expression [6], [7].

The cytokine IL-21 is a member of the common γ chain (γ_c_) family of cytokines, which includes IL-2, IL-4, IL-7 and IL-15 that are involved in T cell proliferation and homeostasis [8]. For example, IL-21 is produced by multiple CD4^+^ T cell subsets including, follicular helper T (T_FH_) cells [9], [10], and was originally described as a cytokine that regulates immunoglobulin production [11]. It is now recognized that the functions of IL-21 also include the induction of IL-10 and IL-17 by CD4^+^ T cells [6], [7], [12], [13], [14], and it is an important factor for the development of T_FH_ cells [15], [16]. However, there are reports that IL-21 is unable to induce the expression of Bcl-6 [17], a transcription factor critical for T_FH_ cell differentiation, and *Il21* ^−/−^ and *Il21r* ^−/−^ mice have normal or near normal levels of T_FH_ cells following viral infection [18], or immunization with sheep red blood cells, ovalbumin, or 4(hydroxy-3-nitrophenyl)acetyl coupled to keyhole limpet hemocyanin [19], [20], [21]. While IL-21 may not be necessary for differentiation of T_FH_ cells, it is essential to the function of these cells as T_FH_ cell-derived IL-21 is crucial for maintenance of germinal center (GC) B cells [19], [21], [22], [23]. In contrast to the role of IL-21 in the regulation of humoral immunity, IL-21 has been shown to be critical for controlling chronic viral infections, particularly LCMV, due to its ability to maintain an effective anti-viral CD8^+^ T cell response [18], [24], [25].

Prior studies have established that *Il21r* ^−/−^ mice infected with *T. gondii* survive for at least 100 days post-infection, yet these mice display a defect in serum IgG [11]. Additionally, IL-21 has been associated with the differentiation of IL-10 producing CD4^+^ T cells [6], [7], which contribute to limiting immune-mediated pathology during toxoplasmosis. However, questions remain about the role of IL-21 in promoting IL-10 and antibody production. Therefore, to elucidate the function of IL-21 in antibody production, CD8^+^ T cell responses, and regulation of the immune response after infection, *Il-21* ^−/−^ mice were challenged with *T. gondii*. In this study, IL-21 deficient mice chronically infected with *T. gondii* have increased numbers of parasites in the brain associated with a decrease in parasite-specific antibody production and a marked reduction in the numbers of effector CD4^+^ and CD8^+^ T cells in the brain, resulting in diminished IFN-γ production. Furthermore, no immunopathology was apparent in *Il21* ^−/−^ mice over the course of the infection, nor was there a defect in development of IL-10^+^ CD4^+^ T cells in chronically infected *Il-21* ^−/−^ mice. Together, these studies indicate the importance of IL-21 in maintaining a T_H_1 effector T cell response in the brain during chronic infection.

## Results

### IL-21 is Required to Control Chronic Infection with *T.* *gondii*

**Figure 1:**
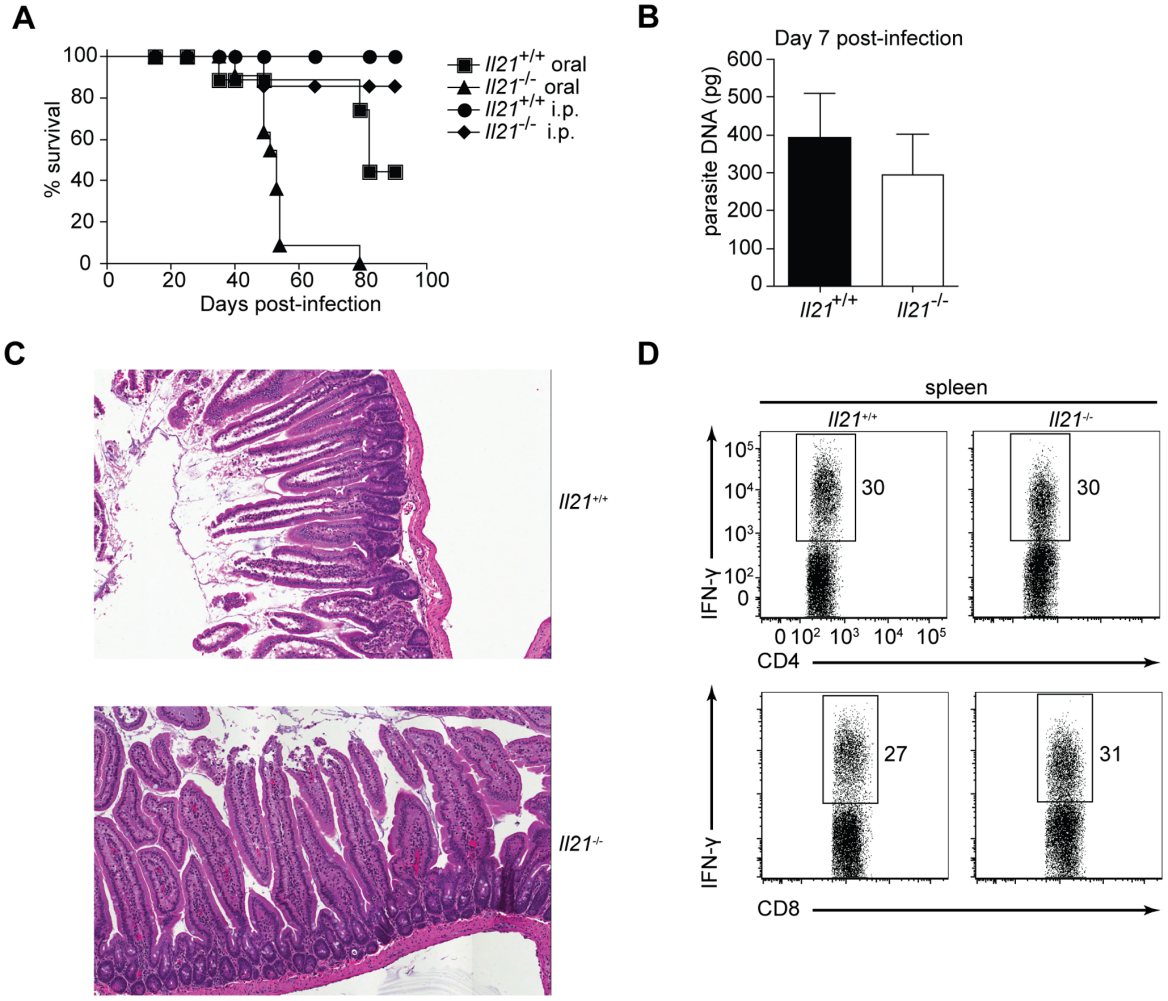
IL-21 is required for resistance to oral infection with *T. gondii*. A) Survival of *Il21* ^−/−^ mice (*n* = 11) and their wild-type littermates (WT; *n* = 7) infected orally, or intraperitoneally (*Il21* ^−/−^ *n* = 6 and WT *n* = 6) with 20 cysts from the ME49 strain of *T. gondii.* B) Quantitative real-time PCR of parasite DNA isolated from the small intestine of WT and *Il21* ^−/−^ mice infected orally with *T. gondii* for seven days. Results are representative of two experiments with three or four mice per group. Error bars represent SEM. C) Histopathology of the small intestine of WT and *Il21* ^−/−^ mice infected orally for twelve days, analyzed by staining with haematoxylin and eosin. Original magnification, ×20. D) Intracellular staining for IFN-γ^+^ CD4^+^ and CD8^+^ T cells from the spleen of WT and *Il21* ^−/−^ mice infected orally for seven days, after stimulation for 4 h *ex vivo* with PMA and ionomycin in the presence of brefeldin A. Numbers outside the boxed areas indicate percent IFN-γ^+^ CD4^+^ (top row) or CD8^+^ T cells (bottom row). Data are representative of two independent experiments with similar results.

**Figure 2:**
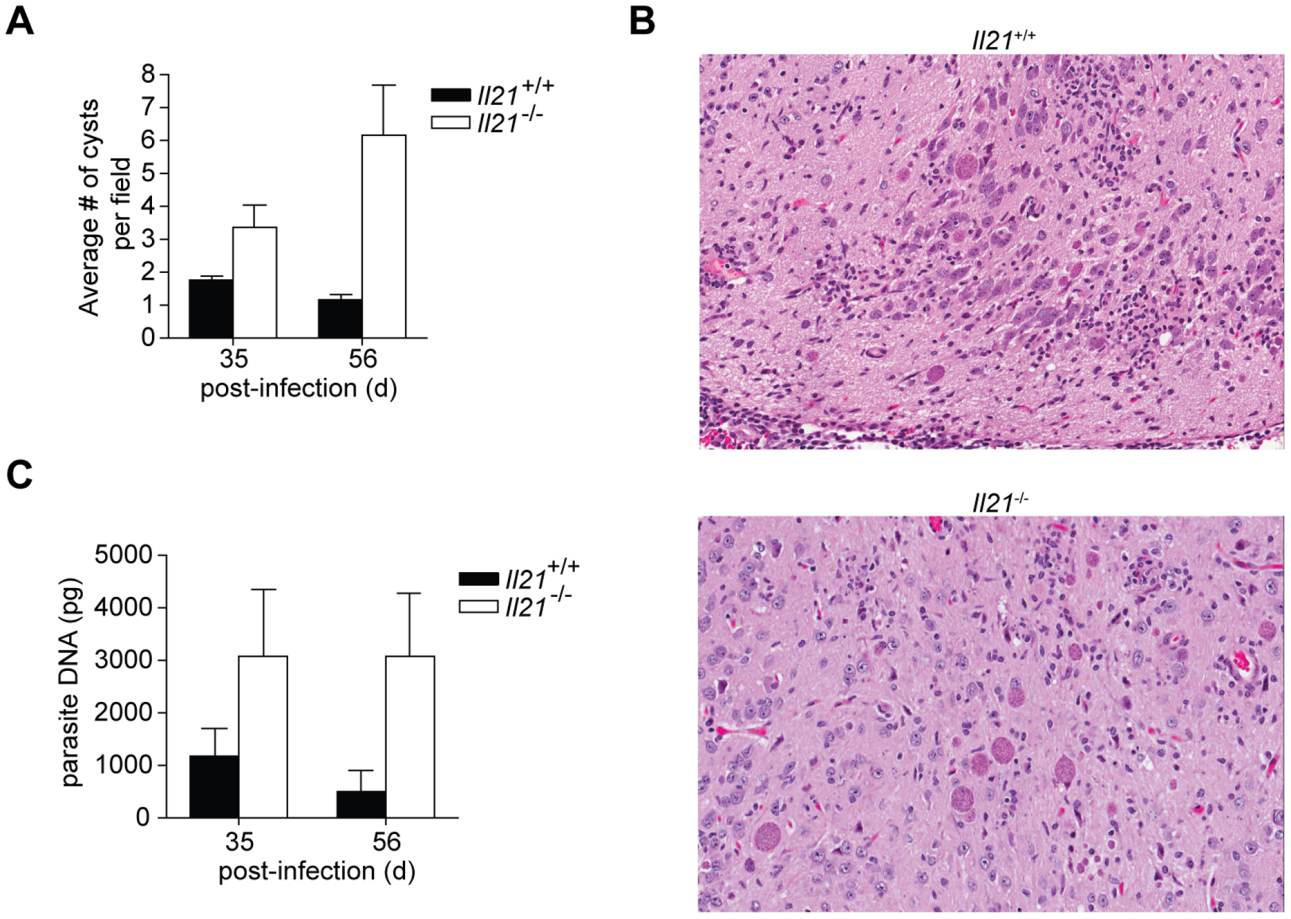
Increased parasite burden in the brain of chronically infected *Il21* ^−/−^ mice. A) Quantitation of the number of ME49 cysts per field in the brain parenchyma of wild-type (WT; *n* = 10) and *Il21* ^−/−^ (*n* = 12) mice at day 35 and 56 after oral infection. Results are representative of six fields per mouse brain section. Error bars represent SEM. B) Histopathology of the brain of orally infected WT and *Il21* ^−/−^ mice at day 56 post-infection, analyzed by staining with haematoxylin and eosin. Original magnification, ×20. C) Quantitative real-time PCR of parasite DNA isolated from the brain of WT and *Il21* ^−/−^ mice at day 35 and 56 after oral infection. Results are representative of three experiments with three to four mice per group. Error bars represent SEM.

In light of recent findings describing the involvement of IL-21 in GC reactions, IL-10 production by CD4^+^ T cells, and in maintenance of CD8^+^ T cell responses, experiments were performed to determine if IL-21 was involved in these processes during *T. gondii* infection. Similar to results observed with *Il21r* ^−/−^ mice [11], intraperitoneal infection of *Il21* ^−/−^ mice with cysts from the ME49 strain of *T. gondii* did not result in increased susceptibility to acute or chronic disease over the time course examined (Fig. 1A). However, when *Il21* ^−/−^ mice were orally infected with ME49 cysts mice survived acute infection, but succumbed to chronic infection between 50-60 days post-infection (Fig. 1A). While no difference in parasite burden, pathology in the gut or the immune response was apparent during the acute response (Fig. 1B–D), histological examination of the brain revealed an increase in the number of cysts/field in *Il21* ^−/−^ mice compared to wild-type littermate controls (Fig. 2A, B). This difference in parasite burden was confirmed by real-time PCR, which indicated that by day 35 post-infection there was an elevated amount of parasite DNA in the brain of *Il21* ^−/−^ mice compared to wild-type mice, which remained elevated at day 56 (Fig. 2C). Together these results suggest that production of IL-21 is important for control of parasite replication in the brain during chronic TE.

### Expression of IL-21 and IL-21r during *Toxoplasma* Infection

**Figure 3:**
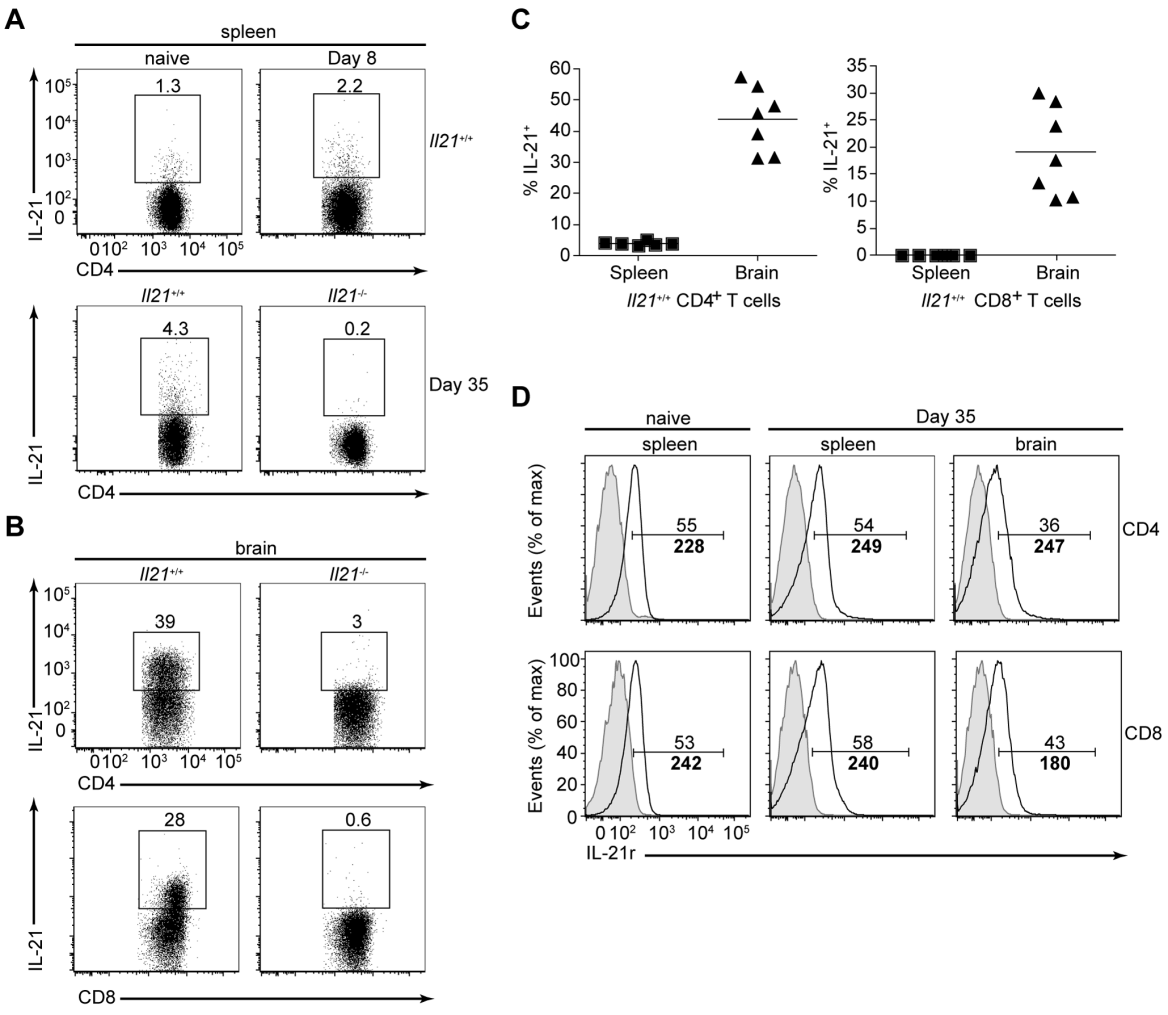
IL-21 and IL-21r expression patterns during chronic toxoplasmic encephalitis. A) Flow cytometry of splenic CD4^+^ T cells from naïve or wild-type (WT) mice infected for 8 or 35 days as well as *Il21* ^−/−^ mice infected for 35 days; splenocytes were stimulated for 4 h *ex vivo* with PMA and ionomycin in the presence of brefeldin A, and stained intracellularly for IL-21. Numbers outside boxed areas indicate percent IL-21^+^CD4^+^ T cells. B) Flow cytometry of BMNCs isolated from WT and *Il21* ^−/−^ mice infected for 35 days; cells were stimulated for 4 h *ex vivo* with PMA and ionomycin in the presence of brefeldin A, and stained intracellularly for IL-21. Numbers outside the boxed areas indicate percent IL-21^+^CD4^+^ T cells (top row) or IL-21^+^CD8^+^ T cells (bottom row). C) The percentage of CD4^+^ (left) and CD8^+^ (right) T cells expressing the cytokine IL-21 in the spleen and brain of individual WT mice infected for 35 days. D) Flow cytometry of splenocytes from naïve (left column) and WT mice infected for 35 days (center column), and BMNCs (right column) isolated from WT mice infected for 35 days; cells were surface stained *ex vivo* to detect IL-21r expression (unshaded histograms). The shaded histogram represents the PE FMO sample for each tissue. Numbers above line indicate percent IL-21r^+^CD4^+^ (top row) or IL-21r^+^CD8^+^ (bottom row) T cells while numbers below line indicate MFI. Data are representative of two independent experiments with similar results.

Given that CD4^+^ and CD8^+^ T cells are a source of IL-21 [26], expression of this cytokine by these populations in the spleen and brain was examined after infection. In the spleen of naïve wild-type mice a small percentage of CD4^+^ T cells were positive for intracellular IL-21, and expression of this cytokine was increased in acutely and chronically infected mice (Fig. 3A). However, IL-21^+^ CD8^+^ T cells were not detected in the spleen at any time point during infection (data not shown). In contrast, a larger proportion of CD4^+^ T cells were found to express IL-21 in the brain of chronically infected wild-type mice, and examination of CD8^+^ T cells revealed a large proportion of these cells are positive for IL-21 (Fig. 3B, C), indicating that these cells gain the ability to produce this cytokine within the brain. No, IL-21 expression was found in T cells derived from the spleen or brain of IL-21 deficient mice confirming specific detection of IL-21 in wild-type mice (Fig. 3A, B).

Next, to determine if T cells in the brain were capable of responding to IL-21 the expression of its cognate receptor was examined. IL-21r expression was detected on a proportion of CD4^+^ and CD8^+^ T cells in the spleen of naïve mice, and expression of this receptor was maintained on T cells during chronic infection (Fig. 3D). Also, IL-21r expression was detected on infiltrating CD4^+^ and CD8^+^ T cells in the brain during chronic TE (Fig. 3D). Comparison of IL-21r expression on T cells from the spleen and brain of chronically infected mice indicated that a smaller percentage of CD4^+^ and CD8^+^ T cells in the brain express the receptor and that the mean fluorescence intensity (MFI) for IL-21r was reduced on CD8^+^, but not CD4^+^ T cells (Fig. 3D). It is plausible that reduction in IL-21r expression on T cells in the brain reflects downregulation of the receptor as part of a negative feedback loop following IL-21 stimulation; however, IL-21r expression on CD4^+^ and CD8^+^ T cells in the spleen and brain of *IL21* ^−/−^ mice resembled that observed for wild-type T cells (data not shown). Regardless, these results indicate that IL-21 is actively secreted during *T. gondii* infection, particularly in the brain, and that effector T cells (CD44^high^CD62L^low^, data not shown) produce and are capable of responding to this cytokine.

### IL-21 is Required to Maintain Germinal Centers during Chronic *T. gondii* Infection

**Figure 4:**
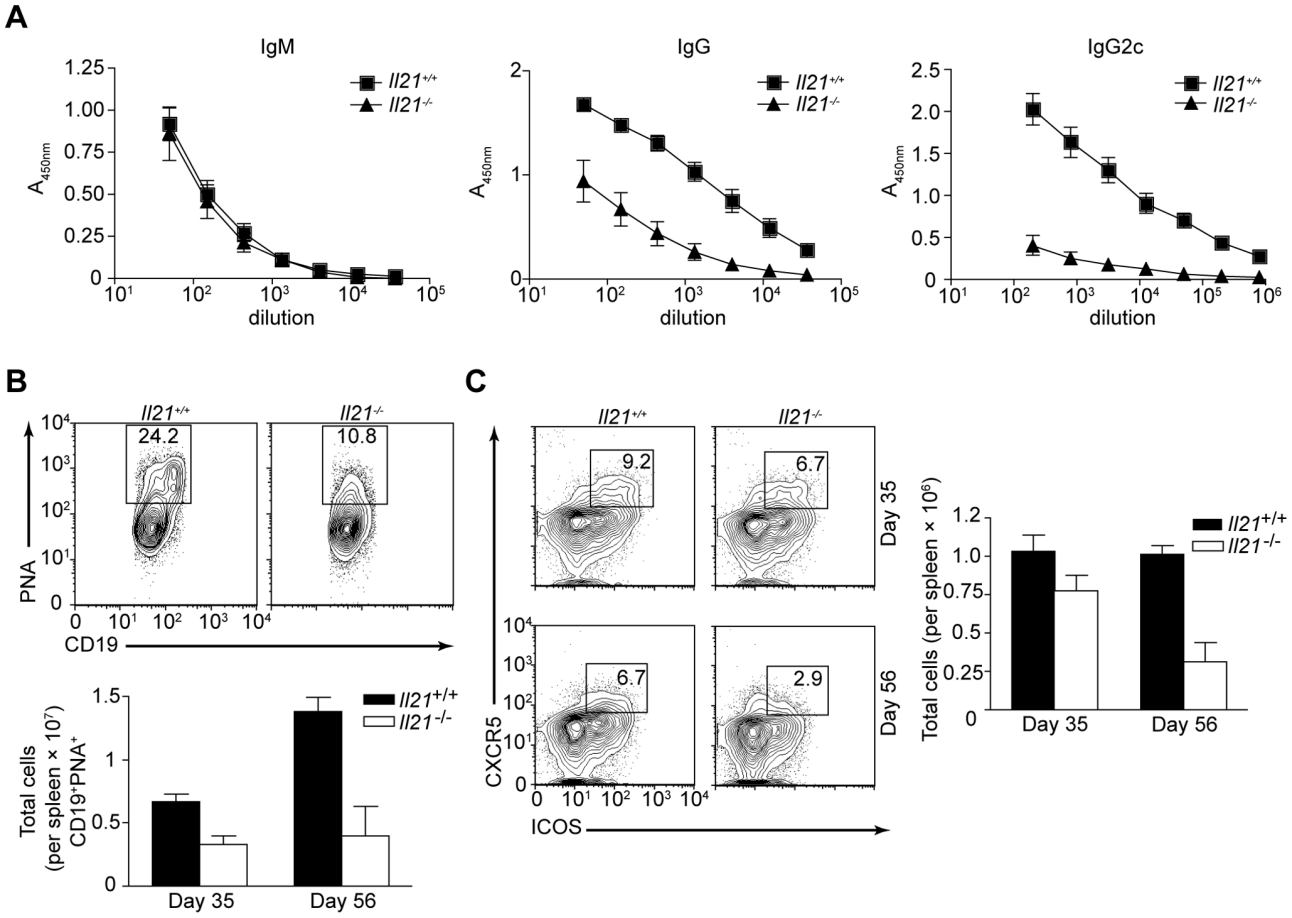
*Il21* ^−/−^ mice have an impaired antibody-mediated immune response during chronic toxoplasmic encephalitis. A) ELISA of IgM, IgG and IgG2c STAg-specific Ab titers in the serum of WT and *Il21* ^−/−^ mice infected for 56 days. B, C) Flow cytometry of splenocytes isolated from day 35 infected WT or *Il21* ^−/−^ mice and stained for (B) germinal center (GC) B cells (CD19^+^B220^+^PNA^+^) or (C) T_FH_ cells (CD4^+^ICOS^+^CXCR5^+^). Numbers in the box represent the percent of (B) PNA^+^CD19^+^ B cells or (C) CD4^+^ICOS^+^CXCR5^+^ T_FH_ cells. Quantitation of (B) GC B cells, or (C) T_FH_ cells on day 35 and 56 p.i. from the spleen of WT and *Il21* ^−/−^ mice. Error bars represent SEM. Data are representative of two (A) or three (B, C) independent experiments with similar results.

Several recent studies have highlighted the involvement of IL-21 in antibody production and proposed that IL-21 contributes to differentiation of T_FH_ cells, and is essential for GC B cell survival and proliferation [17]. Therefore, to determine if there are any defects in the antibody-mediated immune response following *T. gondii* infection STAg-specific IgM and IgG production was measured in the serum of chronically infected wild-type and *Il21* ^−/−^ mice. While no defect in STAg-specific IgM was apparent, *Il21* ^−/−^ mice had a reduction in STAg-specific IgG, including cytophilic IgG2c (Fig. 4A). A defect in IgG production and not IgM is consistent with a role for IL-21 in thymus-dependent (TD) antibody production. As TD antibodies are primarily derived from GC reactions, GC B cells were examined at day 35 post-infection. Defined by PNA^+^ staining, GC B cells were detectable in the spleen of chronically infected *Il21* ^−/−^ mice; however, the percentage and number of GC B cells was reduced compared to their wild-type littermates (Fig. 4B). Furthermore, while the number of PNA^+^ B cells in the spleen increased in wild-type mice over the course of the infection *Il-21* ^−/−^ mice showed only a marginal gain (Fig. 4B).

To determine if an altered T_FH_ cell compartment accompanied the defect in GC B cell numbers in the absence of IL-21, the T_FH_ cell response was evaluated over the course of the infection. While there was no difference in the proportion or number of T_FH_ cells, defined as CD4^+^ICOS^+^CXCR5^+^, at day 35 post-infection, or at any other time point examined earlier in the infection (data not shown), the percentage and number of these cells in the spleen decreased in *Il21* ^−/−^ mice as the infection progressed to day 56 post-infection (Fig. 4C). These findings indicate that IL-21 is not absolutely required for development of T_FH_ cells, but may be necessary for either maintaining this population of T cells, or that the observed reduction in GC B cell numbers is the cause of this late decrease in T_FH_ cell numbers in *Il21* ^−/−^ mice [27].

### The Role of IL-21 in the Production of IL-10 during TE

**Figure 5:**
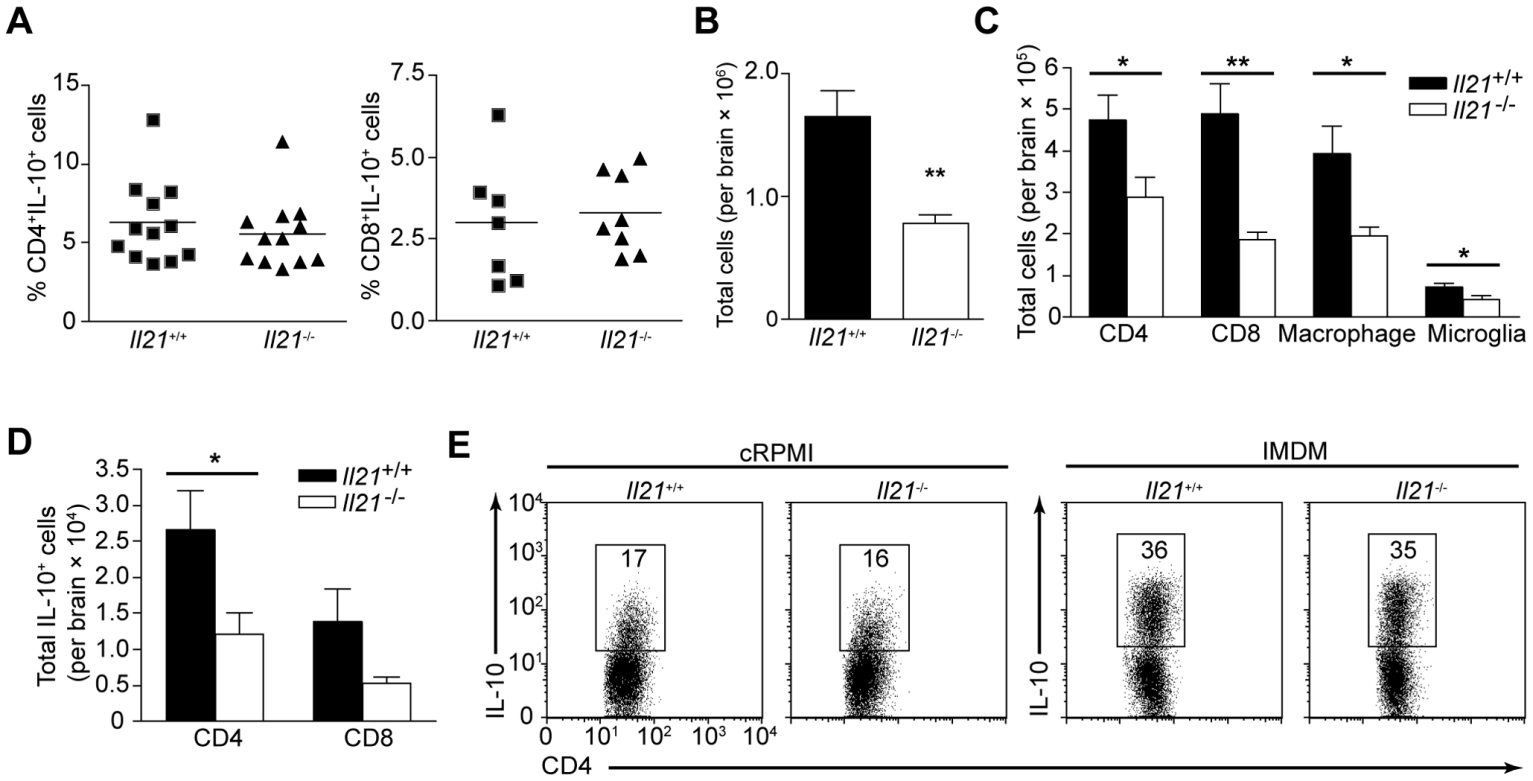
*Il21* ^−/−^ mice have reduced inflammatory cell numbers in the brain during chronic *T. gondii* infection. A) The percentage of CD4^+^ (left) and CD8^+^ (right) T cells expressing the cytokine IL-10 in the brain of individual WT and *Il21* ^−/−^ mice infected for 35 days. Data are representative of two or three independent experiments with three to four mice per group. B) Enumeration of total cell numbers recovered from the brain of WT and *Il21* ^−/−^ mice infected for 35 days. Data are representative of three experiments with similar results. C) Total CD4^+^ T cells, CD8^+^ T cells, macrophages and microglia in each BMNC preparation from (B), calculated by the percentages determined by flow cytometry. D) Total IL-10^+^ CD4^+^ and CD8^+^ T cells in each BMNC preparation from (A), calculated by the percentages determined by flow cytometry. E) Flow cytometry of CD4^+^ T cells isolated from WT and *Il21* ^−/−^ mice, and activated with anti-CD3 and anti-CD28 in cRPMI (left) or IMDM (right) media in the presence of IL-27 for 4 days. Cells were then stimulated for 4 h with PMA and ionomycin in the presence of brefeldin A before staining for intracellular IL-10. Numbers in boxed areas indicate percent IL-10^+^CD4^+^ T cells. Results are representative of three independent experiments with similar results. Significance was determined by a two-tailed unpaired Student’s *t* test. Error bars represent SEM. *P<0.05, **P<0.01.

The production of IL-10 in the brain is critical for prevention of immune-mediated pathology during chronic TE and limiting accumulation of mononuclear cells within the brain [4]. Since IL-21 is implicated in the induction of IL-10 by CD4^+^ T cells [6], [7], we sought to determine if IL-21 is required for production of IL-10 by T cells during chronic TE and if it impacted the overall amount of inflammation. When BMNCs were isolated from chronically infected wild-type and *I121* ^−/−^ mice, and were examined for their ability to make IL-10, no difference in the percentage of IL-10^+^ CD4^+^ or CD8^+^ T cells was seen (Fig. 5A), suggesting that IL-21 is not required to induce IL-10 production by T cells in this setting. Moreover, while there was no decrease in splenocyte numbers (data not shown) absence of IL-21 led to a significant decrease in the number of mononuclear cells (*P* = 0.0005) recovered from the brain of *Il21* ^−/−^ mice (Fig. 5B), including CD4^+^ (*P* = 0.0215) and CD8^+^ (*P* = 0.0003) T cells (Fig. 5C). Additionally, there was a significant decrease in the number of infiltrating macrophages (CD45^+^CD11b^high^) (*P* = 0.0149) and microglia (CD45^+^CD11b^low^) (*P* = 0.0321) by day 35 post-infection (Fig. 5C). Furthermore, as the infection progressed the number of total cells recovered from the brain of *Il21* ^−/−^ mice continued to decline compared to their wild-type littermates (data not shown). The diminished BMNC numbers in *Il21* ^−/−^ mice translated into a significant reduction in the number of IL-10^+^ CD4^+^ T cells (*P* = 0.0326) and a decrease in CD8^+^IL-10^+^ T cells in the brain (Fig. 5D). While this result indicates that fewer CD4^+^ and CD8^+^ T cells are capable of producing IL-10 this did not result in increased inflammation in the brain of *Il21* ^−/−^ mice as opposed to the excessive immune-mediated pathology observed at this site in *Il27ra* ^−/−^ and *Il10* ^−/−^ mice with TE [4], [28].

IL-27 can promote the development of IL-10 producing CD4^+^ T cells, and as mentioned, the absence of IL-27 signaling significantly reduces the number of IL-10–producing T cells in the brain during TE [5]. Several studies indicate that IL-21 is necessary for promoting IL-10 production downstream of T cell activation, or stimulation with IL-27 [6], [7], yet the above findings contrast with these reports. Therefore, to directly determine if IL-21 is involved in induction of IL-10 by IL-27, CD4^+^ T cell-enriched splenocytes from wild-type and *Il21* ^−/−^ mice were activated with antibody to CD3 (anti-CD3) plus anti-CD28 in the presence of IL-27 under non-polarizing conditions (anti-IFN-γ and anti-IL-4) in either complete RPMI (cRPMI) or IMDM. The latter media was chosen because IMDM contains natural agonists for the aryl hydrocarbon receptor (AhR) [29], and activation of AhR has been shown to enhance IL-27 induced IL-10 [30]. Surprisingly, under these conditions absence of IL-21 did not impair the ability of IL-27 to induce IL-10 in either cRPMI or IMDM (Fig. *5*E). Additionally, no difference in IL-10 was measured in the supernatant of wild-type and IL-21 deficient splenocytes stimulated with IL-27 using either medium, thus these results mirror that of the intracellular stain (data not shown).

### Loss of IL-21 Leads to Reduced T Cell-mediated IFN-γ Production during Chronic TE

**Figure 6:**
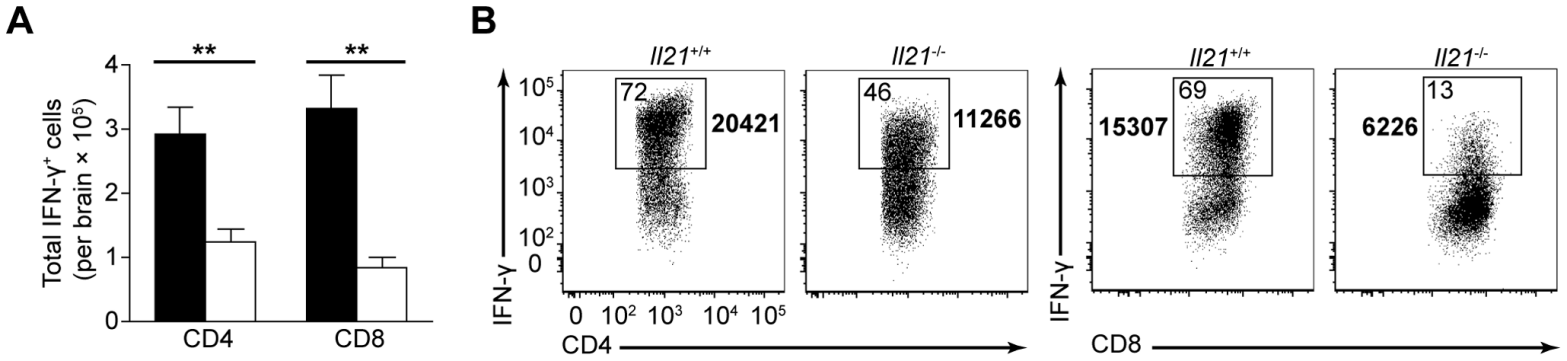
T cells from the brain of *Il21* ^−/−^ mice produce less IFN-γ during chronic toxoplasmic encephalitis. A) Flow cytometry of BMNCs from WT and *Il21* ^−/−^ mice infected for 35 days; cells were stimulated *ex vivo* with PMA and ionomycin in the presence of brefeldin A for 4 h and then stained intracellularly for IFN-γ. Numbers in boxed areas represent percent IFN-γ^+^ CD4^+^ (left) or CD8^+^ (right) T cells, while bold numbers indicate MFI. B) Total IFN-γ^+^CD4^+^ or CD8^+^ T cells isolated from BMNC preparations of WT (black bars) and *Il21* ^−/−^ (white bars) mice infected for 35 days, calculated from the percentages determined by flow cytometry. Data are representative of four independent experiments with similar results. Error bars represent SEM. Significance was determined by a two-tailed unpaired Student’s *t* test. **P<0.01.

The data in Figure 1 shows that *Il-21^−/−^* mice have an elevated parasite burden in the brain. Since the ability of T cells to control *T. gondii* in the brain is dependent on production of IFN-γ [2] studies were performed to determine if the T cell response in the brain of *Il21* ^−/−^ mice is impaired. Though IL-17^+^ T cells make up a small percentage of the total population, there was no observed difference in IL-17 production by T cells in the brain of wild-type and *Il21* ^−/−^ mice (data not shown). Not surprisingly, the reduced population of T cells recovered from the brain of *Il21* ^−/−^ mice translated into a significant decrease in the number of IFN-γ^+^ CD8^+^ (*P*<0.0001), and CD4^+^ T cells (*P* = 0.0009) compared to wild-type mice (Fig. 6A). However, there was also a decrease in the percentage of IFN-γ^+^ CD8^+^ and CD4^+^ T cells in the brain of *Il21* ^−/−^ mice, and the MFI for IFN-γ was also reduced (Fig. 6B). These data suggest that a loss of CD4^+^ and CD8^+^ T cells capable of producing IFN-γ in the brain of *Il21* ^−/−^ mice contribute to the increased parasite burden.

### Effects of IL-21 on Long-lived T Cell Responses

IL-21 has been suggested to play a role in the generation of CD8^+^ T cell memory [31]. Therefore, in order to rule out a requirement for IL-21 in the generation or maintenance of a memory CD8^+^ T cell response wild-type and *Il-21* ^−/−^ mice were infected with a replication deficient strain of *T. gondii* expressing ovalbumin (ΔCPSII-OVA) and then challenged 30 days later with RH-OVA parasites. In this model of immunization it has previously been shown that CD8^+^ T cells are essential for survival against challenge with RH-OVA parasites [32]. Similar numbers of OVA-tetramer^+^ CD8^+^ memory T cells were observed in wild-type and *Il-21* ^−/−^ mice after challenge with the ΔCPSII-OVA parasites, and these mice were able to survive challenge with lethal RH-OVA parasites (data not shown), indicating that IL-21 is not required for generation or maintenance of memory CD8^+^ T cells.

**Figure 7:**
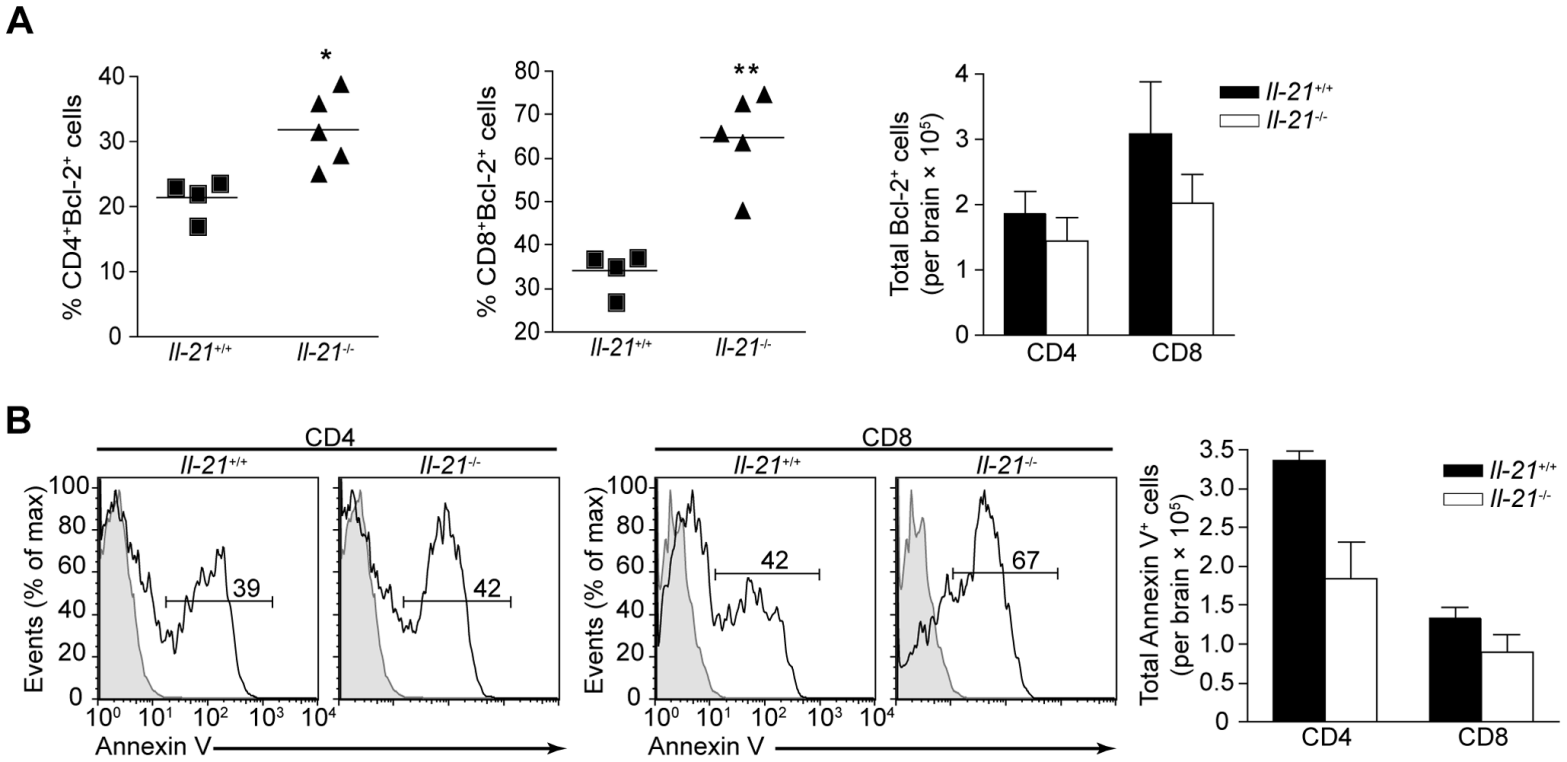
Increased apoptosis of CD8^+^ T cells in the brain of *Il21* ^−/−^ mice during chronic toxoplasmic encephalitis. A) The percentage of CD4^+^ (left) and CD8^+^ (right) T cells expressing Bcl-2 in the brain of individual WT and *Il21* ^−/−^ mice infected for 35 days. Bar graph represents the total number of Bcl-2^+^ CD4^+^ and CD8^+^ T cells in the brain of WT and *Il21* ^−/−^ mice infected for 35 days. B) Flow cytometry of BMNCs from WT and *Il21* ^−/−^ mice infected for 35 days and then stained for the early apoptotic marker Annexin V. Numbers above line indicate percent Annexin V^+^CD4^+^ (left) or Annexin V^+^CD8^+^ (right) T cells in the brain of chronically infected WT and *Il21* ^−/−^ mice. Bar graph represents the total number of Annexin V^+^ CD4^+^ and CD8^+^ T cells in the brain of WT and *Il21* ^−/−^ mice infected for 35 days. Data are representative of two independent experiments with similar results. Error bars represent SEM. Significance was determined by a two-tailed unpaired Student’s *t* test. *P<0.05, **P<0.01.

A possible explanation for the reduction in T cell numbers in the brain of *Il-21* ^−/−^ mice may be an increase in cell death as IL-21 has previously been shown to upregulate anti-apoptotic proteins such as Bcl-2 and Bcl-x_L_ [33], [34]. Intracellular staining for Bcl-2 protein in T cells isolated from the brain of chronically infected wild-type and *Il-21* ^−/−^ mice indicated that a higher proportion of CD4^+^ (*P* = 0.0136) and CD8^+^ (*P* = 0.0010) T cells from IL-21 deficient mice are Bcl-2^+^, resulting in no significant disparity in the total numbers of Bcl-2^+^ CD4^+^ or CD8^+^ T cells (Fig. 7A). As another correlate to determine if the absence of IL-21 leads to an increase in programmed cell death splenocytes and BMNCs were stained for annexin V, a marker of early apoptosis. A comparison of annexin V staining in the spleen of chronically infected mice revealed no difference between the percentage and number of T cells undergoing apoptosis in wild-type and *Il-21* ^−/−^ mice (data not shown). While there was no variance in the percentage of CD4^+^ T cells that stained positive for annexin V there was increased expression by CD8^+^ T cells in IL-21 deficient mice, consequently a similar number of CD8^+^ T cells are undergoing apoptosis in the brain of *Il-21* ^−/−^ mice compared to their wild-type littermates, even though there are less total CD8^+^ T cells (Fig. 7B). These results suggest that apoptosis is occurring at an increased rate in CD8^+^ T cells, but not CD4^+^ T cells in the brain of IL-21 deficient mice.

## Discussion

The previous studies that *Il21r* ^−/−^ mice infected with *T. gondii* survive to 100 days post-infection without showing any sign of increased susceptibility [11], are incongruous with a role for IL-21 in the induction of IL-10, T_FH_ cell development or B cell responses [15], [16], [19], [21], [22], as well as the maintenance of effector CD8^+^ T cell responses [18], [24], [25]. Therefore, experiments were performed to reevaluate the function of IL-21 during toxoplasmosis. The finding that *Il21* ^−/−^ mice are susceptible to chronic toxoplasmosis is more consistent with current models of the role of IL-21. One possible explanation for the different outcomes using *Il21r* ^−/−^ and *Il21* ^−/−^ mice may be the route of infection used, as oral infection may have had an affect on dissemination of the parasite as well as priming of the acute response.

Nevertheless, there are similarities with the previous report using *Il21r* ^−/−^ mice [11]. There was a pronounced reduction in IgG production in the serum of chronically infected *Il21* ^−/−^ mice, particularly STAg-specific IgG2c. The finding that *Il21* ^−/−^ mice were able to generate some class-switched antibodies indicates that other factors, such as IL-6, may also be involved in this process [35]. The reduction in STAg-specific IgG appeared to be a reflection of the loss of GC B cells, rather than impairment in differentiation of T_FH_ cells in the absence of IL-21. Indeed, expression of Bcl-6, a transcription factor required for differentiation of T_FH_ cells, was not reduced in these cells from *Il21* ^−/−^ mice (data not shown). Nor, was any difference in T_FH_ cell numbers noted until well after a decrease in the GC B cell population had occurred; thus, these results are similar to reports that concluded that T_FH_ cell development in *Il21* ^−/−^ and *Il21r* ^−/−^ mice immunized with a number of model antigens or after acute LCMV infection is largely intact [18], [19], [20], [23], and emphasize the importance of IL-21 in maintaining GC B cells [19], [21], [23]. Additionally, the decline in T_FH_ cell numbers in the spleen of *Il21* ^−/−^ mice at day 56 post-infection suggests that loss of IL-21 as a B cell survival factor indirectly impacts survival of T_FH_ cells, potentially through disruption of cognate interactions between these two cell types that are necessary to sustain T_FH_ cells. While *T. gondii* is an obligate intracellular parasite, several studies have identified requirements for B cells in mediating protective immunity against this pathogen [3], [36], [37]. Thus, our finding that a reduction in parasite-specific antibody is accompanied by an increase in parasite burden in the absence of IL-21 lends support to a functional role of parasite-specific antibody in protection against this parasite.

How IL-21 is able to shape effector T cell responses is not fully understood. It has been proposed that IL-21 in combination with other signals, may regulate terminal differentiation and memory T cell formation through modification of the Bcl-6–Blimp-1 axis, as Blimp-1 expression is associated with terminal differentiation and reduced proliferation while Bcl-6 expression can promote survival, proliferation and memory differentiation [38]. Thus, acquisition of IL-21 production by CD4^+^ and CD8^+^ T cells in the spleen or brain during chronic *Toxoplasma* infection, along with loss or gain of additional undefined signals, may favor differentiation of T cells towards a phenotype that produces high amounts of IFN-γ, and can be continually sustained during chronic infection in order to control parasite burden.

In addition to its stimulatory effects on B cell and CD8^+^ T cell proliferation, IL-21 has been linked to production of the anti-inflammatory cytokine IL-10, specifically IL-27 induced IL-10 [6], [7]. While there is evidence that IL-21 can induce IL-10 by T cells [7] it is unclear if this is a direct effect on IL-10 transcription, or a secondary effect of its ability to promote expansion of IL-10 producing T cells. Furthermore, as IL-21 transcription occurs downstream of IL-6 and IL-27 signaling [6], [12], [13], [14], the importance of this cytokine as an immune-regulator may be more pertinent to augmenting or sustaining production of IL-10 rather than initiation of this response. Also, the finding in which CD4^+^ T cells cultured in the presence of antigen-presenting cells do not require autocrine IL-21 in order to induce IL-10 production following IL-27 signaling, suggests that another signal, possibly through a cell ligand-receptor complex like ICOSL-ICOS [6], or a different cytokine may contribute to an enhancement of IL-10, and highlights the presence of multiple pathways that induce IL-10. Moreover, while the number of IL-10 producing T cells is reduced in the brain of *Il-21* ^−/−^ mice during chronic TE the frequency of IL-10^+^ CD4^+^ T cells, including IFN-γ^+^IL-10^+^ double producers (data not shown), is normal, emphasizing that this cytokine is not absolutely required for IL-10 production by T cells. Rather, these studies have indicated an essential role for IL-21 in production of protective anti-parasite specific antibody through preservation of GC B cells, and maintenance of a localized T_H_1 response in the brain during chronic *T. gondii* infection.

## Materials and Methods

### Ethics Statement

All animal studies were carried out in compliance with the guidelines of the Institutional Animal Care and Use Committee (IACUC) of the University of Pennsylvania and in accordance with the recommendations in the Guide and Use of Laboratory Animals of the National Institutes of Health. The animal protocol was approved by the IACUC of the University of Pennsylvania, Philadelphia, PA (Multiple project assurance # A3079-01).

### Mice and Parasites

*Il21* ^+/−^ (B6;129S5-Il21^tm1Lex^/Mmcd) mice were purchased from MMRRC (UC Davis) and bred to generate IL-21–deficient mice and wild-type littermate controls. Mice were housed and bred in specific pathogen–free facilities in the Department of Pathobiology at the University of Pennsylvania in accordance with institutional guidelines.

The ME49 strain of *T. gondii* was prepared from chronically infected CBA/ca mice and experimental animals were infected intraperitoneally or orally with 20 cysts. Mice were monitored daily throughout the infection. Mice were euthanized by CO_2_ inhalation during the chronic infection if they showed any of the following symptoms: cachexia, dehydration, reduced motor function or activity. Soluble toxoplasma antigen (STAg) was prepared from tachyzoites of the RH strain as described previously [39]. For histology small intestines and brains were collected from mice, fixed in 10% formalin, were embedded in paraffin, sectioned and stained with haematoxylin and eosin. For measurement of parasite burden in the small intestine and brain, the 35-fold repetitive *T. gondii B1* gene was amplified by real-time PCR with Power SYBR Green PCR Master mix (Applied Biosystems, Foster City, CA) in an AB7500 fast real-time PCR machine (Applied Biosystems) using published conditions [4].

### Antibodies and Staining Procedures for Brain Mononuclear Cells (BMNC)

BMNCs from chronically infected wild-type and *Il21* ^−/−^ mice were isolated in accordance with a published protocol [4]. Live cells were assessed by preincubation with AmCyan LIVE/DEAD Fixable Dead Cell Stain (Invitrogen, Carlsbad, CA). In order to assess *ex vivo* intracellular cytokine production cells from the spleen and brain were surface and intracellularly stained as previously described [40]. Additionally, intracellular IL-21 was measured by staining first with a recombinant mouse IL-21R subunit Fc chimera protein (R & D systems, Minneapolis, MN) followed by staining with PE–conjugated anti-human IgG (Fcγ-specific) (eBioscience). Annexin V staining was performed as outlined using the annexin V staining kit from BD Biosciences (San Jose, CA). Samples were run immediately after labeling with a FITC–conjugated anti-annexin V antibody. Flow cytometry assays used the following antibodies: FITC–conjugated annexin V, FITC–conjugated Bcl-2, PE–conjugated IL-21R, PE-Cy7–conjugated IFN-γ, APC–conjugated IL-10 (BD Biosciences), and eFluor450–conjugated CD4, PerCp-Cy5.5–conjugated CD8, APC-eFluor780–conjugated CD8, APC-eFluor780–conjugated CD62L, PE-Cy7–conjugated CD44 (eBioscience). Samples were acquired on a BD CantoII (BD Biosciences) and results were analyzed using FlowJo software (Treestar, Ashland, OR).

### Analysis of Splenic GC B Cells and Follicular Helper T Cells

Live cells derived from the spleen were assessed by preincubation with AmCyan LIVE/DEAD Fixable Dead Cell Stain (Invitrogen) prior to surface staining. Antibodies used for staining included Fluorescin–labeled peanut agglutinin (PNA) (Vector Laboratories, Burlingame, CA), FITC–conjugated anti-ICOS, biotin–labeled anti-CXCR5, PerCp-Cy5.5–conjugated anti-CD4 (BD Biosciences), APC-eFluor780 anti-B220/CD45R, eFluor450–conjugated CD19 and APC–conjugated streptavidin (eBioscience).

### T Cell Differentiation

CD4^+^ T cells were isolated from splenocyte samples and lymph nodes that were depleted of CD8^+^ and NK1.1^+^ cells to enrich for CD4^+^ T cells by magnetic bead separation (Polysciences, Niles, IL). Cells were plated in 96-well round-bottom plates (BD Biosciences) at a density of 5×10^6^ cells per ml in either cRPMI or IMDM (Invitrogen). Cells were stimulated with anti-CD3 (1 µg/ml; eBioscience) and anti-CD28 (1 µg/ml; eBioscience). Additionally, IFN-γ and IL-4 were neutralized in all cultures with anti-IFN-γ (10 µg/ml; XMG1.2, eBioscience) and anti-IL-4 (10 µg/ml; 11B11; NCI Preclinical repository). For production of IL-10^+^ T cells, cultures were supplemented with recombinant mouse IL-27 (50 ng/ml; Amgen, Thousand Oaks, CA). CD4^+^ T cells were supplemented with fresh medium and reagents on day 3 and were collected on day 4. T cells were restimulated with PMA and ionomycin plus brefeldin A (Sigma-Aldrich, St. Louis, MO) prior to intracellular staining. Cells were stained using the following antibodies: PerCp–conjugated anti-CD4, PE–conjugated anti-CD8, and APC–conjugated anti-IL-10 (BD Biosciences). A FACSCalibar (BD Biosciences) was used for flow cytometry, and data were analyzed with FlowJo software (Treestar).

### Statistics

Unpaired Student’s *t*-tests were used to determine significant differences, and *P* values less than 0.05 were considered significant.
